# Supplementary material for: Prevalence of opportunistic bacterial infections (tuberculosis and pneumonia) among people with HIV in Ethiopia: Systematic review and meta-analysis
Source: PLoS One. 2025 Oct 21;20(10):e0315599. doi: 10.1371/journal.pone.0315599 (PMC12539741; doi:10.1371/journal.pone.0315599)
Supplement: S2 Table — Comprehensive search strategy for common opportunistic bacterial infections among people living with HIV in Ethiopia. (DOCX) [file pone.0315599.s002.docx]

**S2 Table. Comprehensive search strategy for common opportunistic bacterial infections among people living with HIV in Ethiopia.**

| **Databases** | **Key search terms or phrases** | **Result** |
| --- | --- | --- |
| **PubMed** | **Filters: Humans: Language: English; Date range: 14 December 2013 to 02 May 2023.**  ("Prevalence"[Title]) OR (“Epidemiology” [Title]) AND ("opportunistic infections"[Title/Abstract]) OR ("opportunistic bacterial infections"[Title/Abstract]) OR ("HIV"[Title/Abstract]) OR ("People with HIV "[Title/Abstract]) AND (("Ethiopia"[MeSH Terms]) OR ("Ethiopia"[Title/Abstract])) | 222 |
| **ScienceDirect** | **Filters: Humans: Language: English; Date range: 14 December 2013 to 02 May 2023.**  "Opportunistic infections" "people with HIV/AIDS" "Ethiopia" | 40 |
| **Cochrane Library** | **Filters: Humans: Language: English; Date range: 14 December 2013 to 02 May 2023.**  "Opportunistic infections" "HIV" "Ethiopia" | 4 |
| Total articles retrieved from all databases | 266 |  |
