# Supplementary material for: Prevalence of opportunistic bacterial infections (tuberculosis and pneumonia) among people with HIV in Ethiopia: Systematic review and meta-analysis
Source: PLoS One. 2025 Oct 21;20(10):e0315599. doi: 10.1371/journal.pone.0315599 (PMC12539741; doi:10.1371/journal.pone.0315599)
Supplement: S3 Table — The quality of included studies was evaluated using the Joanna Briggs Institute (JBI) quality appraisal checklist for prevalence studies. (DOCX) [file pone.0315599.s003.docx]

**S3 Table. Quality appraisal result of included studies. The quality of included studies was evaluated using the Joanna Briggs Institute (JBI) quality appraisal checklist for prevalence studies.**

| **Study** | **1. Was the sample frame appropriate to address the target population?** | **2. Were study participants sampled in an appropriate way?** | **3. Was the sample size adequate?** | **4. Were the study subjects and the setting described in detail?** | **5. Was the data analysis conducted with sufficient coverage of the identified sample?** | **6. Were valid methods used for the identification of the condition?** | **7. Was the condition measured in a standard, reliable way for all participants?** | **8. Was there appropriate statistical analysis?** | **9. Was the response rate adequate, and if not, was the low response rate managed appropriately?** | **Score (percentile)** | **Overall appraisal:** |
| --- | --- | --- | --- | --- | --- | --- | --- | --- | --- | --- | --- |
| Damtie *et al*., 2013 [20] | Yes | Yes | Yes | Yes | Yes | Yes | Yes | No | Yes | 8 (88.9) | Included |
| Moges & Kassa, 2014 [21] | Yes | Yes | Yes | Yes | Yes | Yes | Yes | Yes | Yes | 9 (100) | Included |
| Mitku *et al.*, 2015 [22] | Yes | Yes | Yes | Yes | Yes | Yes | No | No | Yes | 7(77.8) | Included |
| Alemayehu *et al.,* 2017 [23] | Yes | Yes | Yes | Yes | Yes | Yes | Yes | Yes | Yes | 9 (100) | Included |
| Deribe & Estifanos, 2018 [24] | Yes | Yes | Yes | Yes | Yes | Yes | Yes | Yes | Yes | 9 (100) | Included |
| Solomon *et al.* 2018 [25] | Yes | Yes | Yes | Yes | Yes | Yes | Yes | No | Yes | 8 (88.9) | Included |
| Urgessa *et al*. 2018 [26] | Yes | Yes | Yes | Yes | Yes | Yes | No | Yes | Yes | 8 (88.9) | Included |
| Dereje *et al.* 2019 [27] | Yes | Yes | Yes | Yes | Yes | Yes | Yes | Yes | Yes | 9 (100) | Included |
| Melkamu *et al.* 2020 [28] | Yes | Yes | Yes | Yes | Yes | Yes | Yes | Yes | Yes | 9 (100) | Included |
| Fite & Aga, 2020 [29] | Yes | Yes | Yes | Yes | Yes | Yes | Yes | No | Yes | 8 (88.9) | Included |
| Wachamo & Bonja, 2020 [30] | Yes | Yes | Yes | Yes | Yes | Yes | Yes | Yes | Yes | 9 (100) | Included |
| Weldearegawi *et al.* 2020 [31] | Yes | Yes | Yes | Yes | Yes | Yes | Yes | Yes | Yes | 9 (100) | Included |
| Tegegne *et al*. 2020 [32] | Yes | Yes | Yes | Yes | Yes | Yes | Yes | Yes | Yes | 9 (100) | Included |
| Chanie *et al*. 2021 [33] | Yes | Yes | Yes | Yes | Yes | Yes | Yes | No | Yes | 8 (88.9) | Included |
| Dembelu & Wosenelh, 2021 [34] | Yes | Yes | Yes | Yes | Yes | Yes | Yes | Yes | Yes | 9 (100) | Included |
| Kebede *et al.* 2022 [35] | Yes | Yes | Yes | Yes | Yes | Yes | Yes | Yes | Yes | 9 (100) | Included |
| Mequanente *et al.* 2022 [36] | Yes | Yes | Yes | Yes | Yes | Yes | Yes | Yes | Yes | 9 (100) | Included |
| Birmeka, 2023 [37] | Yes | Yes | Yes | Yes | Yes | Yes | Yes | No | Yes | 8 (88.9) | Included |
| Dagnaw *et al*. 2023 [38] | Yes | Yes | Yes | Yes | Yes | Yes | Yes | Yes | Yes | 9 (100) | Included |
| Mekonnen *et al.* 2023 [39] | Yes | Yes | Yes | Yes | Yes | Yes | Yes | Yes | Yes | 9 (100) | Included |

**References**

20. Damtie D, Yismaw G, Woldeyohannes D, Anagaw B. Common opportunistic infections and their CD4 cell correlates among HIV-infected patients attending at antiretroviral therapy clinic of Gondar University Hospital, Northwest Ethiopia. BMC research notes. 2013;6:1-7. <https://doi.org/10.1186/1756-0500-6-534> PMID: 24330921

21. Moges N, Kassa G. Prevalence of opportunistic infections and associated factors among HIV positive patients taking anti-retroviral therapy in DebreMarkos Referral Hospital, Northwest Ethiopia. J AIDs Clin Res. 2014;5(5):1-300. <https://doi.org/10.4172/2155-6113.1000301>

22. Mitiku H, Weldegebreal F, Teklemariam Z. Magnitude of opportunistic infections and associated factors in HIV-infected adults on antiretroviral therapy in eastern Ethiopia. HIV/AIDS-Research and Palliative Care. 2015:137-44. <https://doi.org/10.2147/HIV.S79545> PMID: 25999763

23. Alemayehu M, Yisehak Y, Alaro W, Alemayehu B. Opportunistic infections among HIV/AIDS patients taking ante-retroviral therapy at tertiary care hospital in Wolaita zone, southern Ethiopia. J AIDS Clin Res. 2017;8(2):1-4. <https://doi.org/10.4172/2155-6113.1000665>

24. Deribe A, Estifanos W. Magnitude and Determinants of Opportunistic Infections Among HIV/AIDS Patients in Sphmmc, Addis Ababa, Ethiopia, Retrospective Study. JOJ Public Health. 2018;4:555627. <https://doi.org/10.19080/JOJPH.2018.04.555627>

25. Solomon FB, Angore BN, Koyra HC, Tufa EG, Berheto TM, Admasu M. Spectrum of opportunistic infections and associated factors among people living with HIV/AIDS in the era of highly active anti-retroviral treatment in Dawro Zone hospital: a retrospective study. BMC research notes. 2018;11:1-7. <https://doi.org/10.1186/s13104-018-3707-9> PMID: 30126450

26. Urgessa F, Ararsa A, Ataro Z. Prevalence and associated risk factors of opportunistic infections among anti-retro viral treatment naive HIV/AIDS infected patients. J AIDS Clin Res. 2018;9(03). <https://doi.org/10.4172/2155-6113.1000763>

27. Dereje N, Moges K, Nigatu Y, Holland R. Prevalence And predictors of opportunistic infections among HIV positive adults on antiretroviral therapy (On-ART) versus Pre-ART In Addis Ababa, Ethiopia: A comparative cross-sectional study. HIV/AIDS-Research and Palliative Care. 2019:229-37. <https://doi.org/10.2147/HIV.S218213> PMID: 31632155

28. Melkamu MW, Gebeyehu MT, Afenigus AD, Hibstie YT, Temesgen B, Petrucka P, et al. Incidence of common opportunistic infections among HIV-infected children on ART at Debre Markos referral hospital, Northwest Ethiopia: a retrospective cohort study. BMC infectious diseases. 2020;20:1-12. <https://doi.org/10.1186/s12879-020-4772-y> PMID: 31948393

29. Fite MB, Aga DJ. Spectrum of Opportunistic Disease and Associated Factors among Patients Attending ART Clinic, Nekemte Specialized Hospital, Western Ethiopia. bioRxiv. 2020:2020.10. 06.327668. <https://doi.org/10.1101/2020.10.06.327668>

30. Wachamo D, Bonja F. Magnitude of opportunistic infections and associated factors among HIV-positive adults on art at selected public hospitals in Sidama National Regional State, Southern Ethiopia. HIV/AIDS-Research and Palliative Care. 2020:479-87. <https://doi.org/10.2147/HIV.S265274> PMID: 33061659

31. Weldearegawi TZ, Gerensea H, Berihu H, Gidey G, Welearegay MZ. The magnitude of opportunistic infections and associated factors in HIV-infected adults on antiretroviral therapy in southern zone Tigray, Ethiopia: a cross-sectional study. Pan African Medical Journal. 2020;35(1). <https://doi.org/10.11604/pamj.2020.35.126.17839> PMID: 32637024

32. Dagnaw Tegegne K, Cherie N, Tadesse F, Tilahun L, Kassaw MW, Biset G. Incidence and predictors of opportunistic infections among adult HIV infected patients on anti-retroviral therapy at Dessie comprehensive specialized hospital, Ethiopia: a retrospective follow-up study. HIV/AIDS-Research and Palliative Care. 2022:195-206. <https://doi.org/10.2147/HIV.S346182> PMID: 35469324

33. Chanie ES, Bayih WA, Birhan BM, Belay DM, Asmare G, Tiruneh T, et al. Incidence of advanced opportunistic infection and its predictors among HIV infected children at Debre Tabor referral Hospital and University of Gondar Compressive specialized hospitals, Northwest Ethiopia, 2020: A multicenter retrospective follow-up study. Heliyon. 2021;7(4). <https://doi.org/10.1016/j.heliyon.2021.e06745> PMID: 33912717

34. Dembelu M, Kote M, Gilano G, Mohammed T. Incidence and predictors of reoccurrence of opportunistic infection among adult HIV/AIDS patients attending ART clinic at public health facilities in Arba Minch town, southern Ethiopia: A retrospective cohort study. PloS one. 2021;16(12):e0261454. <https://doi.org/10.1371/journal.pone.0261454> PMID: 34972122

35. Kebede N, Ambaye T, Bazie GW. Opportunistic Infections and Associated Factors Among HIV-Infected Adults on Antiretroviral Therapy Attending Health Facilities in North Wollo, Ethiopia.

36. Mequanente D, Srinivasan P, Mallika G, Thamimul Ansari P, Wale M. Incidence of Opportunistic Infections among HIV-infected Children on ART at Gondar University Specialized Hospital, Ethiopia. Indian Journal of Science and Technology. 2022;15(34):1675-82. <https://doi.org/10.17485/IJST/v15i34.1073>

37. Birmeka M. Distribution pattern and prevalence of opportunistic infections and their possible reciprocal effects among HIV patients, Burayu health centers, Ethiopia. The Journal of Infection in Developing Countries. 2023;17(07):1022-9. <https://doi.org/10.3855/jidc.15105> PMID: 37515807

38. Dagnaw M, Fekadu H, Gebre Egziabher A, Yesfue T, Indracanti M, Tebeje A. Incidence of opportunistic infections and its predictors among HIV/AIDS patients on antiretroviral therapy in Gondar University Comprehensive and Specialized Hospital, Ethiopia. HIV Research & Clinical Practice. 2023;24(1):2187013.

39. Mekonnen GB, Birhane BM, Engdaw MT, Kindie W, Ayele AD, Wondim A. Predictors of a high incidence of opportunistic infections among HIV-infected children receiving antiretroviral therapy at Amhara regional state comprehensive specialized hospitals, Ethiopia: a multicenter institution-based retrospective follow-up study. Frontiers in Pediatrics. 2023;11:1107321. <https://doi.org/10.3389/fped.2023.1107321> PMID: 37205221
