# Supplementary material for: Prevalence of opportunistic bacterial infections (tuberculosis and pneumonia) among people with HIV in Ethiopia: Systematic review and meta-analysis
Source: PLoS One. 2025 Oct 21;20(10):e0315599. doi: 10.1371/journal.pone.0315599 (PMC12539741; doi:10.1371/journal.pone.0315599)
Supplement: Attachment — Submitted filename: Reviewed3062024.docx [file pone.0315599.s004.docx]

Title: Prevalence and trends of opportunistic bacterial infections (Tuberculosis and pneumonia) among HIV positive patients in Ethiopia: Systematic review and meta-analysis

Manuscript Number: PONE-D-24-22113

My comments and concerns are as follow:

**Major**

The focus of MS: Trends vs Systematic review & meta-analysis

The trends are not indicated properly.

What is the clear definition of pneumonia? Not clear and requires definition

If bacteriology confirmed pneumonia, the prevalence of predominant isolates should be reported.

How retrospective studies included in this study showed/confirmed the pneumonia?

**Minor**

Population: mainly focus on children, better to balance and align with objective of the study and title.

Is the study protocol registered in PRESPOROUS? If so, indicate the registration number

Language: better to use people/person first language. Thus, use ‘**people with HIV**’ than HIV positive patients.

I suggest the revisions of conclusion and recommendations according to the objective and title of the study and main findings.

Good luck
