# Supplementary material for: Prevalence of opportunistic bacterial infections (tuberculosis and pneumonia) among people with HIV in Ethiopia: Systematic review and meta-analysis
Source: PLoS One. 2025 Oct 21;20(10):e0315599. doi: 10.1371/journal.pone.0315599 (PMC12539741; doi:10.1371/journal.pone.0315599)
Supplement: Attachment — Submitted filename: Review report_Melese Abate Reta.docx [file pone.0315599.s005.docx]

**Date: 11 August 2024**

**REVIEW REPORTS**

**PLOS ONE**

**Title:** Prevalence and trends of opportunistic bacterial infections (Tuberculosis and pneumonia) among HIV positive patients in Ethiopia: Systematic review and meta-analysis.

**MS #:** PONE-D-24-22113

**Dear Editor,**

Thank you for inviting me to review this manuscript. The topic is both intriguing and essential, as it plays a vital role in understanding the dynamics of opportunistic infections in HIV-positive individuals. By assessing the prevalence of these infections, healthcare providers can gain valuable insights into the factors contributing to disease progression in these patients. This understanding is crucial for developing targeted interventions, optimizing treatment plans, and enhancing diagnostic accuracy. Ultimately, such research contributes to more effective patient management, reducing the morbidity and mortality associated with HIV and improving the overall quality of life for those affected by the virus. However, the study does have major limitations. Please see my observations and comments for each section of the document below.

**COMMENTS**

**TITLE:**

Prevalence and trends of opportunistic bacterial infections (Tuberculosis and pneumonia) among HIV positive patients in Ethiopia: Systematic review and meta-analysis

**Comments:** Since this study did not evaluate the trends of opportunistic infections (such as TB and pneumonia) among HIV-positive individuals, it would be inappropriate to include "trends of" in your title. Please remove "trend" from the title.

**ABSTRACT**

**Conclusions: “**This meta-analysis shows that the trend of tuberculosis infection is rising, then

falling, then rising again, while the trend of pneumonia infection among HIV-positive individuals

in Ethiopia is rising”.

**Comments:** If you’re certain that a trend analysis was conducted, please include the results in the abstract. However, I didn’t see this information in the results section.

**Keywords: “** Ethiopia, meta-analysis, prevalence, pneumonia, tuberculosis, systemic review, and opportunistic infection”.

**Comments:** Better if you can make like

HIV(+)-people, opportunistic infections, tuberculosis, pneumonia, prevalence, systematic review, meta-analysis, Ethiopia

**INTRODUCTION:**

**Comments:** The introduction section does not adequately address Ethiopian studies on the prevalence of opportunistic infections, such as TB and pneumonia, among HIV-positive individuals in Ethiopia. Please revise the introduction to include this discussion.

AIDS

**Comments:** Please first provide the full definition of any term, followed by its abbreviation in parentheses, the first time it is mentioned in the document. After that, you may use the abbreviation consistently throughout the rest of the document.

“A 2019 report states that OI was the main cause of nearly 95,000 child deaths from

HIV-related causes [3].”

**Comments:** A 2019 global report…

“There were 310,000 OI-related fatalities among AIDS patients in the eastern and southern African regions, even though there is insufficient information about the OIs' recurrence rate in the continent [6].

**Comment:** please check the reference. "6. Teker AG. AIDS-related deaths in Turkey between 2009 and 2018. Epidemiology & Infection. 2021;149:e191."

“Globally, 9.9 million cases of tuberculosis and 214,000 HIV-positive deaths are expected in 2021, according to WHO estimates [9].”

**Comments:** Please review this sentence for accuracy, or consider using the most recent data.

“ Ethiopia is one of the 30 countries with a high TB and TB/HIV burden; with an estimated annual TB incidence of 140/100,000 persons and a death rate of 19 per 100,000 people, according to the 2020 Global TB Report [9]”.

**Comments:** Please refer to the updated WHO report on Ethiopia's TB burden, either the 2022 or 2023 edition.

**METHODS**

**2. 1 Country profile**

**Comments:** I believe the country profile information is not particularly relevant to this manuscript. Instead, it would be more appropriate for the authors to focus on the burden of HIV, TB, pneumonia, and other opportunistic infections in Ethiopia, and include related data that is pertinent to the manuscript.

If you believe the country profile is relevant to the manuscript, please revise it to include the most up-to-date information and provide appropriate citations or references.

**2.2 Search strategy**

….. ‘‘opportunistic bacterial infections in Ethiopia’’ ‘‘Prevalence of opportunistic bacterial

infections in HIV positive patients,’’ ‘‘Prevalence of opportunistic bacterial infections in Ethiopia,’’and ‘‘Magnitude of opportunistic bacterial infections among HIV positive patients in different regions of Ethiopia’’ were used to search journals.”

**Comments:** Please provide the search strategy you followed and the total number of articles captured in at least one database (e.g., PubMed) as a supplementary file. Ensure the information is formatted appropriately, either using "AND" or "OR" operators, or with commas, as needed.

……………..and study locations (conducted between September 14/2023 and December 1, 2023),

**Comments:** The above sentence is not clear, and needs revision

**2.3 Inclusion and exclusion criteria of the studies**

**Comments:** Please change with “eligibility criteria”

**2.3.1 Inclusion criteria**

**Comments:** This paragraph does not outline the inclusion criteria for potential papers in this systematic review and meta-analysis. Instead, it discusses the characteristics of the included studies. Please clearly state your inclusion criteria: specify which types of papers and necessary information were considered for inclusion, and detail which papers and types of information were deemed irrelevant or excluded.

**2.3.2 Exclusion criteria**

**Comments:** Please explicitly list all of your exclusion criteria.

What about papers that are only available as abstracts, conference presentations, or lack full-text access?

**2.4. Study selection procedures**

**….** An EndNote X7 reference program,

**Comments:** Include the specifications for EndNote.

……inter-rater agreement was calculated after referring to the Cochrane Handbook of Systematic Reviews.

**Comment:** Cite the reference

**2.7 Risk of publication bias**

**Comments:** please remove this subheading and include the infromation (risk of publication bias into 2.8 Data analysis subheading section.

**3. Results**

**Comments**: include a subheading " Searching results"

“A total of 276 articles were retrieved on the prevalence and determinants of OIs among HIVpositive patients were retrieved in Ethiopia”

**Comments**: Assessing the determinants of opportunistic infections among HIV-positive individuals is not the objective of your study. Please ensure that your focus remains on the actual objectives of your research.

“Of the remaining 27 articles, 7 articles were further removed for different purposes (lack of OR, CI and number of positive cases)”.

**Comments:** Why did you exclude articles that lacked OR and CI data, given that your primary outcome of interest is the prevalence of opportunistic infections (TB and pneumonia) among HIV-positive individuals? Since your study does not assess the determinants of these infections, it is unclear why these papers were excluded. Please revise this decision.

“Therefore, 20 of the studies met the eligibility criteria and were included in

the final systematic review and meta-analysis study (Figure 1)”.

**Comments:** Please revise the PRISMA flow chart as follows: Records after duplicates removed (n=151) → Records screened (n=151) → Full-text articles assessed for eligibility (n=27) → Studies included in the systematic review and meta-analysis (n=20).

**3.1 Characteristics of the eligible studies**

**Comments**: The paragraph requires significant editing and language revision.

**Table 1**: Prevalence of tuberculosis among HIV patients in Ethiopia

**Comments:** Characters-tics of included studies to calculate the pooled prevalence of TB among HIV(+)-individuals

**Table 2:** Prevalence of pneumonia among HIV patients in Ethiopia

**Comments**: Characteristics of included studies to calculate the pooled prevalence of pneumonia among HIV(+)-individuals

**3.2** Pooled prevalence of opportunistic bacterial infections

**Comments:** Pooled prevalence of tuberculosis among HIV(+) individuals

**4. Discussion**

**Comments:**

The entire document requires thorough language editing, including improvements in sentence structure, grammar, and overall clarity. Careful attention should be given to revising the text to ensure it is well-constructed, grammatically correct, and easy to understand.

Your study presents a pooled or combined prevalence, which involves aggregating data from multiple sources to provide a more comprehensive and reliable estimate. Given the nature of this approach, it may not be appropriate or recommended to compare your findings with individual primary studies, as these studies typically offer a narrower scope and may not capture the broader trends that your meta-analysis addresses. Instead, it would be more suitable and meaningful to discuss your results in the context of other meta-analyses or national reports. These sources provide a wider perspective and are more comparable to your pooled data, allowing for a more accurate and robust discussion of your findings. By focusing on these broader sources, you can better validate your results and place them within the larger body of evidence.

**5.1 Conclusions**

“In terms of the prevalence of tuberculosis, the pattern is rising, falling, and rising.

However, pneumonia prevalence among HIV positive patients is increasing”.

**Comments:** You did not estimate the pooled prevalence of TB across different time intervals, so this statement is not accurate. Please ensure that your conclusions are precise and based directly on the findings of your study.
