# Supplementary material for: Prevalence of opportunistic bacterial infections (tuberculosis and pneumonia) among people with HIV in Ethiopia: Systematic review and meta-analysis
Source: PLoS One. 2025 Oct 21;20(10):e0315599. doi: 10.1371/journal.pone.0315599 (PMC12539741; doi:10.1371/journal.pone.0315599)
Supplement: Attachment — Submitted filename: Response to Reviewers.docx [file pone.0315599.s007.docx]

Dear reviewers, we are grateful for your time and effort, and all your comments and suggestions are constructive and supportive, which increases the readability and overall quality of the paper. Below, we provide a point-by-point response explaining how we have addressed each of the reviewer's comments and suggestions. N.B., yellow and bright green, highlighted, are the answers for reviewers 1 and 2, respectively, and turquoise color for both reviewers. We hope that the revised manuscript will better fit the journal.

**General Comments to the Author**

**Recommendation 1:** Is the manuscript technically sound, and do the data support the conclusions?

The manuscript must describe a technically sound piece of scientific research with data that supports the conclusions. Experiments must have been conducted rigorously, with appropriate controls, replication, and sample sizes. The conclusions must be drawn appropriately based on the data presented.

**Reviewer #1:** Partly

**Reviewer #2:** No

**Answer 1:** Dear reviewers, Thanks for your valuable recommendation; we accepted it and corrected as per your suggestion.

**Recommendation 2:** Has the statistical analysis been performed appropriately and rigorously?

**Reviewer #1:** Yes

**Reviewer #2:** Yes

**Answer 2:** Dear reviewers, Thanks for your appreciation.

**Recommendation 3:** Have the authors made all data underlying the findings in their manuscript fully available?

The PLOS Data policy requires authors to make all data underlying the findings described in their manuscript fully available without restriction, with rare exception (please refer to the Data Availability Statement in the manuscript PDF file). The data should be provided as part of the manuscript or its supporting information, or deposited to a public repository. For example, in addition to summary statistics, the data points behind means, medians and variance measures should be available. If there are restrictions on publicly sharing data—e.g. participant privacy or use of data from a third party—those must be specified.

**Reviewer #1:** Yes

**Reviewer #2:** Yes

**Answer 3:** Dear reviewers, Thanks for your appreciation.

**Recommendation 4:** Is the manuscript presented in an intelligible fashion and written in standard English?

PLOS ONE does not copyedit accepted manuscripts, so the language in submitted articles must be clear, correct, and unambiguous. Any typographical or grammatical errors should be corrected at revision, so please note any specific errors here.

Reviewer #1: Yes

Reviewer #2: No

**Answer 4:** Dear reviewer 2, Thanks for your valuable recommendation; we accepted it and corrected as per your suggestion.

**Answer for Reviewer 1#**

**Major**

**Recommendation 1:** The focus of MS: Trends vs Systematic review & meta-analysis. The trends are not indicated properly.

**Answer 1:** Dear reviewer, Thanks for your valuable recommendation, but the trend is not relevant and was removed.

**Recommendation 2:** What is the clear definition of pneumonia? Not clear and requires definition.

**Answer 2:** Dear reviewer, Thanks for your valuable recommendation; we accepted it and corrected as per your suggestion. Could you please go to the introduction?

**Recommendation 3:** If bacteriology confirmed pneumonia, the prevalence of predominant isolates should be reported.

**Answer 3:** Dear reviewer, Thanks for your valuable recommendation, but they are not mentioned in the primary studies.

**Recommendation 4:** How retrospective studies included in this study showed/confirmed the pneumonia?

**Answer 4:** Dear reviewer, Thanks for your valuable recommendation; but it was and editorial problem and now amended. Could you please go to the result?

**Minor**

**Recommendation 30:** Population: mainly focus on children, better to balance and align with objective of the study and title.

**Answer 30:** Dear reviewer, Thanks for your valuable recommendation; we accepted it and corrected as per your suggestion. Could you please go to the introduction?

**Recommendation 31:** Is the study protocol registered in PRESPOROUS? If so, indicate the registration number.

**Answer 31:** Dear reviewer, yes it is already registered and found in abstract methodology section.

**Recommendation 32:** Language: better to use people/person first language. Thus, use ‘**people with HIV**’ than HIV positive patients.

**Answer 32:** Dear reviewer, Thanks for your valuable recommendation; we accepted it and corrected as per your suggestion. Could you please go to the entire document of the manuscript?

**Recommendation 33:** I suggest the revisions of conclusion and recommendations according to the objective and title of the study and main findings.

**Answer 33:** Dear reviewer, Thanks for your valuable recommendation; we accepted it and corrected as per your suggestion. Could you please go to the conclusion and recommendation?

**Answer for Reviewer 2#**

**Recommendation 1:** Since this study did not evaluate the trends of opportunistic infections (such as TB and pneumonia) among HIV-positive individuals, it would be inappropriate to include "trends of" in your title. Please remove "trend" from the title.

**Answer 1:** Dear reviewer, Thanks for your valuable recommendation; we accepted it and corrected as per your suggestion. Could you please go to the title and other parts of the manuscript?

**Recommendation 2:** If you’re certain that a trend analysis was conducted, please include the results in the abstract. However, I didn’t see this information in the results section.

**Answer 2:** Dear reviewer, Thanks for your valuable recommendation; we accepted it and corrected as per your suggestion. Could you please go to the abstract?

**Recommendation 3**: Better if you can make the key words like, HIV (+)-people, opportunistic infections, tuberculosis, pneumonia, prevalence, systematic review, meta-analysis, Ethiopia

**Answer 3:** Dear reviewer, Thanks for your valuable recommendation; we accepted it and corrected as per your suggestion. Could you please go to the abstract?

**Introduction:**
**Recommendation 4:** The introduction section does not adequately address Ethiopian studies on the prevalence of opportunistic infections, such as TB and pneumonia, among HIV-positive individuals in Ethiopia. Please revise the introduction to include this discussion.

**Answer 4:** Dear reviewer, Thanks for your valuable recommendation; we accepted it and corrected as per your suggestion. Could you please go to the introduction?

**Recommendation 5:** Please first provide the full definition of any term, followed by its abbreviation in parentheses, the first time it is mentioned in the document. After that, you may use the abbreviation consistently throughout the rest of the document. “A 2019 report states that OI was the main cause of nearly 95,000 child deaths from HIV-related causes [3].”

**Answer 5:** Dear reviewer, Thanks for your valuable recommendation; we accepted it and corrected as per your suggestion. Could you please go to the introduction?

**Recommendation 6:** A 2019 global report… “There were 310,000 OI-related fatalities among AIDS patients in the eastern and southern African regions, even though there is insufficient information about the OIs' recurrence rate in the continent [6].

**Answer 6:** Dear reviewer, Thanks for your valuable recommendation; we accepted it and corrected as per your suggestion. Could you please go to the introduction?

**Recommendation 7:** please check the reference. "6. Teker AG. AIDS-related deaths in Turkey between 2009 and 2018. Epidemiology & Infection. 2021;149:e191." “Globally, 9.9 million cases of tuberculosis and 214,000 HIV-positive deaths are expected in 2021, according to WHO estimates [9].”

**Answer 7:** Dear reviewer, Thanks for your valuable recommendation; we accepted it and corrected as per your suggestion. Could you please go to the introduction?

**Recommendation 8:** Please review this sentence for accuracy, or consider using the most recent data. “Ethiopia is one of the 30 countries with a high TB and TB/HIV burden; with an estimated annual TB incidence of 140/100,000 persons and a death rate of 19 per 100,000 people, according to the 2020 Global TB Report [9]”.

**Recommendation 9:** Please refer to the updated WHO report on Ethiopia's TB burden, either the 2022 or 2023 edition

**Answer 9:** Dear reviewer, Thanks for your valuable recommendation; we accepted it and corrected as per your suggestion. Could you please go to the introduction?

**Methods**

**Recommendation 10:** Country profile: I believe the country profile information is not particularly relevant to this manuscript. Instead, it would be more appropriate for the authors to focus on the burden of HIV, TB, pneumonia, and other opportunistic infections in Ethiopia, and include related data that is pertinent to the manuscript. If you believe the country profile is relevant to the manuscript, please revise it to include the most up to-date information and provide appropriate citations or references.

**Answer 10:** Dear reviewer, Thanks for your valuable recommendation; we accepted it and corrected as per your suggestion. Could you please go to the methodology?

**Recommendation 11:** Please provide the search strategy you followed and the total number of articles captured in at least one database (e.g., PubMed) as a supplementary file. Ensure the information is formatted appropriately, either using "AND" or "OR" operators, or with commas, as needed.

**Answer 11:** Dear reviewer, Thanks for your valuable recommendation; we accepted it and corrected as per your suggestion. Could you please go to the methodology?

**Recommendation 12:** ……………..and study locations (conducted between September 14/2023 and December 1, 2023). The above sentence is not clear, and needs revision.

**Answer 12:** Dear reviewer, Thanks for your valuable recommendation; we accepted it and corrected as per your suggestion. Could you please go to the methodology?

**Recommendation 13:** Please change inclusion and exclusion criteria of the studies with “eligibility criteria”

**Answer 13:** Dear reviewer, Thanks for your valuable recommendation; we accepted it and corrected as per your suggestion. Could you please go to the methodology?

**Recommendation 14:** An Inclusion criterion, this paragraph does not outline the inclusion criteria for potential papers in this systematic review and meta-analysis. Instead, it discusses the characteristics of the included studies. Please clearly state your inclusion criteria: specify which types of papers and necessary information were considered for inclusion, and detail which papers and types of information were deemed irrelevant or excluded.

**Answer 14:** Dear reviewer, Thanks for your valuable recommendation; we accepted it and corrected as per your suggestion. Could you please go to the methodology?

**Recommendation 15:** Exclusion criteria, please explicitly list all of your exclusion criteria. What about papers that are only available as abstracts, conference presentations, or lack full-text access?

**Answer 15:** Dear reviewer, Thanks for your valuable recommendation; we accepted it and corrected as per your suggestion. Could you please go to the methodology?

**Recommendation 16:** Study selection procedures …. An EndNote X7 reference program, Include the specifications for EndNote. ……inter-rater agreement was calculated after referring to the Cochrane Handbook of Systematic Reviews. Cite the reference

**Answer 16:** Dear reviewer, Thanks for your valuable recommendation; we accepted it and corrected as per your suggestion. Could you please go to the methodology?

**Recommendation 17:** Risk of publication bias please removes this subheading and includes the information (risk of publication bias into 2.8 Data analysis subheading section.

**Answer 17:** Dear reviewer, Thanks for your valuable recommendation; we accepted it and corrected as per your suggestion. Could you please go to the methodology?

Results

**Recommendation 18:** include a subheading "Searching results" “A total of 276 articles were retrieved on the prevalence and determinants of OIs among HIV positive patients were retrieved in Ethiopia.

**Answer 18:** Dear reviewer, Thanks for your valuable recommendation; we accepted it and corrected as per your suggestion. Could you please go to the result?

**Recommendation 19:** Assessing the determinants of opportunistic infections among HIV-positive individuals is not the objective of your study. Please ensure that your focus remains on the actual objectives of your research. “Of the remaining 27 articles, 7 articles were further removed for different purposes (lack of OR, CI and number of positive cases)”.

**Answer 19:** Dear reviewer, Thanks for your valuable recommendation; we accepted it and corrected as per your suggestion. Could you please go to the result?

**Recommendation 20:** Why did you exclude articles that lacked OR and CI data, given that your primary outcome of interest is the prevalence of opportunistic infections (TB and pneumonia) among HIV- positive individuals? Since your study does not assess the determinants of these infections, it is unclear why these papers were excluded. Please revise this decision. “Therefore, 20 of the studies met the eligibility criteria and were included in the final systematic review and meta-analysis study (Figure 1)”.

**Answer 20:** Dear reviewer, Thanks for your valuable recommendation; we accepted it and corrected as per your suggestion. Could you please go to the result?

**Recommendation 21:** Please revise the PRISMA flow chart as follows: Records after duplicates removed (n=151) → Records screened (n=151) → Full-text articles assessed for eligibility (n=27) → Studies included in the systematic review and meta-analysis (n=20).

**Answer 21:** Dear reviewer, Thanks for your valuable recommendation; we accepted it and corrected as per your suggestion. Could you please go to the result?

Characteristics of the eligible studies

**Recommendation 22:** The paragraph requires significant editing and language revision

**Answer 22:** Dear reviewer, Thanks for your valuable recommendation; we accepted it and corrected as per your suggestion. Could you please go to the result?

**Recommendation 23:** Characteristics of included studies to calculate the pooled prevalence of TB among HIV (+) individuals on the prevalence of tuberculosis among HIV patients in Ethiopia

**Answer 23:** Dear reviewer, Thanks for your valuable recommendation; we accepted it and corrected as per your suggestion. Could you please go to the result?

**Recommendation 24:** Characteristics of included studies to calculate the pooled prevalence of pneumonia among HIV (+)-individuals on prevalence of pneumonia among HIV patients in Ethiopia

**Answer 24:** Dear reviewer, Thanks for your valuable recommendation; we accepted it and corrected as per your suggestion. Could you please go to the result?

**Recommendation 25:** Correct pooled prevalence of opportunistic bacterial infections to pooled prevalence of tuberculosis among HIV (+) individuals.

**Answer 25:** Dear reviewer, Thanks for your valuable recommendation; we accepted it and corrected as per your suggestion. Could you please go to the result?

Discussion

**Recommendation 26:** The entire document requires thorough language editing, including improvements in sentence structure, grammar, and overall clarity. Careful attention should be given to revising the text to ensure it is well-constructed, grammatically correct, and easy to understand. Your study presents a pooled or combined prevalence, which involves aggregating data from multiple sources to provide a more comprehensive and reliable estimate. Given the nature of this approach, it may not be appropriate or recommended to compare your findings with individual primary studies, as these studies typically offer a narrower scope and may not capture the broader trends that your meta-analysis addresses. Instead, it would be more suitable and meaningful to discuss your results in the context of other meta-analyses or national reports. These sources provide a wider perspective and are more comparable to your pooled data, allowing for a more accurate and robust discussion of your findings. By focusing on these broader sources, you can better validate your results and place them within the larger body of evidence.

**Answer 26:** Dear reviewer, Thanks for your valuable recommendation; we accepted it and corrected as per your suggestion. Could you please go to the Discussion?

Conclusions

**Recommendation 27:** “In terms of the prevalence of tuberculosis, the pattern is rising, falling, and rising. However, pneumonia prevalence among HIV positive patients is increasing”. Comments: You did not estimate the pooled prevalence of TB across different time intervals, so this statement is not accurate. Please ensure that your conclusions are precise and based directly on the findings of your study.

**Answer 27:** Dear reviewer, Thanks for your valuable recommendation; we accepted it and corrected as per your suggestion. Could you please go to the Conclusion?
