# Supplementary material for: Prevalence of opportunistic bacterial infections (tuberculosis and pneumonia) among people with HIV in Ethiopia: Systematic review and meta-analysis
Source: PLoS One. 2025 Oct 21;20(10):e0315599. doi: 10.1371/journal.pone.0315599 (PMC12539741; doi:10.1371/journal.pone.0315599)
Supplement: Attachment — Submitted filename: Response to Reviewer 3.docx [file pone.0315599.s008.docx]

Mengistu Hailemariam Zenebe, PhD

Academic Editor

PLOS ONE

November 19, 2024

Dear Dr. Mengistu Hailemariam Zenebe,

We are pleased to submit the revised draft of our manuscript, “Prevalence of opportunistic bacterial infections (tuberculosis and pneumonia) among people with HIV in Ethiopia: Systematic review and meta-analysis” (PONE-D-24-22113R1), to *PLOS ONE*. We appreciate the time and effort dedicated by the editorial staff and reviewers. The comments provided were valuable and helped us refine our paper. As such, we have made several revisions to the manuscript based on the suggestions given. Changes to the manuscript are yellow highlighted.

Below are our point-by-point responses to the reviewers’ comments.

**Response to Reviewer 3**

Thank you for your insightful comments and suggestions. Please find the answers to each of your questions below.

1. **At the very beginning, there is a study conducted previously with the same title and study area and even broader than the current study (prevalence and determinants of opportunistic infections among HIV-infected adults receiving ART in Ethiopia: A systematic review and meta-analysis). If so what is the main reason to conduct this study at the moment for fear of duplication effort?**

Response: Previously, Woldegeorgis and colleagues [14] conducted a systematic review and meta-analysis regarding the prevalence and determinants of opportunistic infections among HIV-infected adults receiving ART in Ethiopia. However, the prevalence of opportunistic bacterial infections, particularly tuberculosis and pneumonia, among people of all age groups living with HIV in the country is not collected, well organised, and documented as a systematic review and meta-analysis. Therefore, the objective of this systematic review and meta-analysis is to estimate the overall prevalence of opportunistic bacterial infections among people living with HIV from available research conducted in different regions of Ethiopia. The findings of this work will be used by the concerned stakeholders to reduce the prevalence of opportunistic bacterial infections and design evidence-based interventions (page 4, last paragraph).

2. **Introduction
   1. The introduction lacks chronological order; it would be better if the authors were encouraged to revise it as the following:
   -Define the problem clearly, and report the burden of the problem from global to national or from developed to developing nations
   -Explain the impacts/consequences of the problem if left unaddressed or untreated
   -The possible risk factors of opportunistic infections in people with HIV (of course this is not your objective you can skip)
   -Finally mention what has been done previously to tackle the problem and gaps your study is going to address. Here boldly show the justification of the current study.
   2. Avoid citing wrong references. For instance, refer. 6
   3. Focus on your objective only (pneumonia among non-RVI children is not objective so avoid it) and the introduction is mostly about pneumonia ignoring tuberculosis why?**

Response: Thank you for your close reading of our paper. This is an important point, so we have amended as per your suggestions (pages 3 & 4).

3. **Methods
   1. In search strategy Google Scholar is not a database remove it.
   2. Merge the inclusion and exclusion criteria into eligibility criteria and almost all articles from Ethiopia published in English. So this shouldn’t be a criteria remove it
   3. Quality appraisal put cut off point of JBI for studies to be included or excluded
   4. Data analysis: what is your base to conclude I2 25% is low and 75% high heterogeneity or p<0.05 significant publication bias? Put citations**

Response: We agree with your suggestion and have modified accordingly (pages 5-8).

4. **Results
   1. Bring the study selection procedure under the result subsection
   2. This has publication so what did you do for that?
   3. To identify the possible source of heterogeneity please consider meta-regression
   4. When conducting subgroup analysis please try to merge smaller numbers into one as others (eg. Tigray region can be merged with Oromia or Amhara in the TB subgroup)
   5. In the figures of sensitivity analysis (fig. 6) the estimates should be the same with pooled prevalence of TB and pneumonia.**

Response: Thank you for this observation our answer for all of your suggestions are found below. This is because (1) study selection process must be included in the method section as per “PRISMA-2020 checklist”. Please check the “Supplemental File 1”. The second (2) comment is not clear. (3) Dear reviewer, regarding identifying the possible source of heterogeneity using meta-regression is optional and our team chooses the figure representation rather than the meta-regression. (4) We did not do it because Tigray is one of the regions found in Ethiopia and merging with Oromia or Amhara region results falsified report or due to this study, stakeholders may not consider Tigray region particularly during reducing the prevalence of opportunistic bacterial infections and design evidence-based interventions. With regard to sensitivity analysis (5) As far as we know, a sensitivity analysis is carried out by removing each study one at a time in order to clarify the impact of each study on the pooled effect size. During the sensitivity analysis, studies not included in Figs 6a & b had relatively determinant effects on the overall magnitude of tuberculosis and pneumonia among people living with HIV in Ethiopia, so we removed them and this is scientifically valid way of reporting the sensitivity analysis. Dear reviewer, in this regard we recommended to see more information from different previously published works elsewhere or get in touch with statistician.

5. **Conclusion and recommendations
   1. Try to conclude based on your study’s findings not based on your general knowledge.
   2. In the recommendation section you mentioned that “Organize regular training sessions for healthcare workers to enhance their knowledge regarding opportunistic infections, diagnostic criteria, and treatment protocols specific to HIV-positive patients. Provide healthcare professionals with updated clinical guidelines, treatment algorithms, and educational resources that focus on the management of opportunistic infections in the context of HIV.” The question does your study reveal that the increment of opportunistic infection among people living with HIV is due to a lack of trained professionals or poor knowledge?**

Response: We agree with your suggestion and have modified accordingly (pages 18 & 19).

6. **Generally, intense language editing is needed throughout the whole manuscript to make it clearer and easily understandable for readers. Please avoid using expressions like “HIV-positive” or “HIV patients” throughout the whole document they seem to be stigmatizing instead replace them with “people living with HIV” or “people with HIV”.**

Response: We agree with your suggestion and have modified accordingly (throughout the manuscript).

Sincerely,

Abayeneh Girma

Corresponding author
